# 

**Published:** 1863-12

**Authors:** 


New Series, Vol. 6, No. 12. Whole Series, Vol. 20, No. 12.

Dr R. C. Hamill
Box 1305

### THE CHICAGO MEDICAL JOURNAL.

DANIEL BRAINARD, M. D.,

J. ADAMS ALLEN, M. D.,

Editors and Proprietors.

DECEMBER, 1863.

CHICAGO:
J. C. W. BAILEY, BOOK AND JOB PRINTER, 130 CLARK STREET.
1863.

